# Graph Attention Informer for Long-Term Traffic Flow Prediction under the Impact of Sports Events

**DOI:** 10.3390/s24154796

**Published:** 2024-07-24

**Authors:** Yaofeng Song, Ruikang Luo, Tianchen Zhou, Changgen Zhou, Rong Su

**Affiliations:** School of Electrical and Electronic Engineering, Nanyang Technological University, Singapore 639798, Singapore; yaofeng.song@ntu.edu.sg (Y.S.); ruikang001@e.ntu.edu.sg (R.L.); zhou0429@e.ntu.edu.sg (T.Z.)

**Keywords:** ITS, deep learning, traffic flow prediction, attention, sports events

## Abstract

Traffic flow prediction is one of the challenges in the development of an Intelligent Transportation System (ITS). Accurate traffic flow prediction helps to alleviate urban traffic congestion and improve urban traffic efficiency, which is crucial for promoting the synergistic development of smart transportation and smart cities. With the development of deep learning, many deep neural networks have been proposed to address this problem. However, due to the complexity of traffic maps and external factors, such as sports events, these models cannot perform well in long-term prediction. In order to enhance the accuracy and robustness of the model on long-term time series prediction, a Graph Attention Informer (GAT-Informer) structure is proposed by combining the graph attention layer and informer layer to capture the intrinsic features and external factors in spatial–temporal correlation. The external factors are represented as sports events impact factors. The GAT-Informer model was tested on real-world data collected in London, and the experimental results showed that our model has better performance in long-term traffic flow prediction compared to other baseline models.

## 1. Introduction

Traffic flow prediction is a core component of an Intelligent Transportation System (ITS) [1]. An ITS integrates people, vehicles, and roads into a comprehensive consideration so that they can work closely together to achieve a synergistic effect [2]. Traffic flow prediction is a rather complex process with multiperiod features, which is affected by a variety of factors such as traffic patterns, abnormal events, bad weather, and data collection [3]. For the government, traffic flow prediction mainly serves at the macro level, such as in urban master planning, urban transportation development policy, and urban transportation professional planning [4]. Accurate and efficient traffic flow prediction helps to alleviate urban traffic congestion and improve urban transportation efficiency [5,6]. For individual travelers, traffic flow prediction is an important reference for path planning [7], which is helpful for people to plan their travel routes in advance to avoid getting into traffic congestion.

In modern society, traffic congestion is a common problem [8,9]. Traffic congestion not only causes inconvenience to people’s traveling, but it also may produce a series of problems for some specific occasions, such as sports events. There have been some reports of teams not being able to get to the stadium on time due to traffic congestion, thus causing games to be postponed. For fans, the delay has forced them to readjust their follow-up plans, and for those who traveled from afar, this may cause more inconvenience. During large-scale sports events, urban traffic will see a surge in urban road traffic and public transport passenger flow [10]. Compared to normal urban traffic planning, the traffic organization during large-scale sports events has unique travel and service characteristics [11]: it requires safe, smooth, reliable, and high-level services for the event traffic and must ensure the normal operation of the host city’s traffic system at the same time.

With the arrival of the era of big data, the development of big data’s enabling of transportation has attracted wide attention [12]. Based on a better traffic flow prediction result, the traffic police can conduct timely traffic diversion to ease congestion caused by excessive traffic. While companies such as cabs, online car-hailing and bike-sharing can conduct vehicle scheduling to safeguard the public’s need for transportation. Short-term prediction provides accurate forecasting from minutes to hours, thus benefiting applications such as real-time traffic control and traveler information systems. This includes practices like adjusting traffic signal timings based on real-time data, rapid response to incidents such as accidents and breakdowns to minimize disruptions, and providing real-time updates and rerouting suggestions through navigation systems. Meanwhile, long-term prediction offers forecasts over weeks to months, which is helpful for developing traffic management plans for special events to handle temporary surges. In this case, how to effectively use big data to make good traffic flow prediction, especially under the impact of special events, has become a new challenge.

In terms of the development process of traffic flow prediction, the methods can be categorized into classical methods and deep learning methods [13]. Among the classical methods, models based on statistical methods have better interpretability, such as the Historical Average Model (HA) [14], the Autoregressive Integrated Moving Average (ARIMA) [15], and the Kalman filter [16]. Models based on traditional machine learning methods are more flexible, such as the Support Vector Machine (SVM) [17], K-Nearest Neighbor (KNN) [18], and Hidden Markov Model (HMM) [19]. However, classical methods are difficult to characterize the deep nonlinear relationships in spatiotemporal series, and they are not ideal for predicting data with high data perturbation. In recent years, with the development of deep learning, more and more researchers tend to consider deep learning methods, such as the Convolutional Neural Network (CNN) [20], Recurrent Neural Network (RNN) [21], Graph Convolutional Network (GCN) [22], Gate Recurrent Unit (GRU) [23], Long Short-Term Memory (LSTM) [24], and their variants. These models perform well in short-term prediction, but their accuracy and stability are not satisfactory for long-term prediction.

Compared to the above analysis, we propose a novel deep neural network model, which can effectively combine the spatiotemporal information of traffic maps and accurately realize long-term traffic flow prediction. The contributions are as follows:

- To the best of our knowledge, this is the first study to consider the impact of major sports events in traffic flow prediction.
- The Graph Attention Informer (GAT-Informer) structure is firstly proposed to address the long-term traffic flow prediction problem by combining the Graph Attention Network and the Informer Network.
- In addition to using the classical dataset, another dataset used to verify and test GAT-Informer is newly collected real-world data containing real sports events. This dataset has not been applied to other published articles.

The rest of the work is organized as follows: Section 2 issues the problem and describes the development of traffic flow prediction methods. Section 3 first gives the quantitative criteria for the impact of sports events on traffic flow; it then follows with the description of the model structure. Section 4 presents the detailed experimental results and the visualization of model performance comparisons with analysis. Section 5 summarizes the conclusions and the outlook for future research.

## 2. Related Research

### 2.1. Problem Statement

Traffic flow is the number of vehicles passing through a spatial unit (e.g., a roadway segment or a traffic detector) in a given time period [25]. Traffic flow prediction can be categorized into short-term prediction and long-term prediction according to the prediction length. Long-term traffic flow prediction corresponds to transportation planning, while short-term traffic flow prediction corresponds to traffic guidance, traffic management, and control. There is no clear criterion for which category it belongs to, and it is generally believed that a prediction length of 5–30 min is short-term, while more than 30 min is long-term [26,27]; some researchers also define 1 day and above as long-term [28]. In this paper, the prediction length of 30 min or less is regarded as a short-term prediction. For a large-scale sports event, the traffic department needs to do traffic planning in advance so as to establish an efficient and coordinated departmental communication and traffic command system to ensure the effective implementation of the various stages of planning and orderly operation.

### 2.2. Classical Prediction Model

Without considering traffic maps, traffic flow prediction problems can be reduced to a time series analysis. ARIMA is the most common time series prediction model in statistical modeling [29]. It contains Autoregressive (AR), Integrated (I), and Moving Average (MA) models. The AR model captures historical trends and makes forecasts accordingly, but it is not ideal to deal with data with sudden changes. The I model performs linear subtraction of data with equal period intervals to smooth the data. The MA model makes up for the shortcomings of the AR model and is used to deal with noisy terms. Many variants of ARIMA have been proposed to continuously optimize the model implementation over time. Seasonal ARIMA [30] supports time series data with seasonal components, Subset ARIMA [31] improves the accuracy of short-term prediction tasks, and FARIMA [32] improves the accuracy of long-term prediction tasks. However, these models require data with high smoothness and a linear relationship, which makes it difficult to deal with data containing special events such as sports events.

### 2.3. Deep Learning-Based Prediction Model

In order to utilize the advantages of each neural network to realize a better prediction performance, a combination of two or more models is generally used. With the proposal of an attention mechanism in the Transformer [33], many researchers applied it to traffic flow prediction. DNN-BTF [7] applies an attention mechanism to automatically learn the importance of past traffic flow information and uses a CNN to mine the spatial features, as well as an RNN to mine the temporal features of traffic flow. GCN-DHSTNet [34] dynamically learns the spatiotemporal features of traffic crowd flow data on a citywide scale and simultaneously predicts the traffic flow in each area. STGCN [26] is the first to apply graph convolution to the traffic prediction problem. Based on this, ASTGCN [35] introduces an attention mechanism to capture spatiotemporal correlations. GMAN [36] predicts the traffic conditions at different locations at different time steps on a road network graph. DMSTGCN [37] devises a dynamic graph constructor and a dynamic graph convolution method to propagate the hidden states of the nodes based on dynamic spatial relationships. STFGCN [38] efficiently learns the hidden spatiotemporal dependencies using a novel fusion operation of various spatial and temporal graphs to process different time steps in parallel.

In conclusion, motivated by related works and existing problems, the Graph Attention Informer (GAT-Informer) network for long-term traffic prediction, which combines both the spatial and temporal correlations of each detector in the traffic communication network while satisfying the high accuracy requirement of long-term time series prediction is proposed in this paper. In the next section, the structure of the entire model will be illustrated in detail.

## 3. Methodology

### 3.1. Problem Formulation

At time t, the input data contain the traffic flow information of each detector, $X_{t}=\left[x_{1},x_{2},\ldots ,x_{n}\right]$, where $X_{t}\in R^{n\times F}$, $x_{a}\in R^{F}$ refers to the *a*th input node with a feature dimension *F*, and *n* is the number of total detectors. A graph is the basic structure of a Graph Neural Network (GNN) [39], and the traffic map is a special type of graph. It can be defined as follows:

(1) G=(V,E,A)

where *V* is the set of nodes, *E* is the set of edges, and *A* is the adjacency matrix.

Node set *V* with *n* nodes contains the traffic flow information for each node, and different nodes are connected by the edge set *E* with a link adjacency matrix *A*. We consider a directed graph, and the node adjacency is described in the adjacency matrix *A* indicating the connectivity between nodes.

The main purpose of the graph-based traffic flow prediction is to predict the future *N* step traffic flow, which can be described as follows:

(2) Y=f(X;G)

(3) $$X={\left[X_{t-M},X_{t-M+1},\ldots ,X_{t}\right]}^{T}$$

(4) $$Y={\left[Y_{t+1},Y_{t+2},\ldots ,Y_{t+N}\right]}^{T}$$

where *X* is the historical traffic flow data, *Y* is the future traffic flow value, *f* is the relationship function, *M* is the time step of historical data usage, and *N* is the prediction horizon.

The effect of sports events is a dynamic factor which variates by time. An impact factor σ is utilized to denote the effect of sports events in the temporal domain. At time step *t*, the impact factor is described as $\sigma_{t}\in R^{n}$ and the *M* steps $\sigma \in R^{M\times n}$. With the consideration of sports events effect, the prediction can be further expressed as:

(5) Y=f(X,σ;G)

(6) $$\sigma ={\left[\sigma_{t-M},\sigma_{t-M+1},\ldots ,\sigma_{t}\right]}^{T}$$

In conclusion, the issue of traffic flow prediction under the impact of sports events is to find the relationship function *f*, which will be realized by our GAT-Informer model. In the next subsection, the mathematical expression of the external factor that we considered in this paper will be introduced in detail.

### 3.2. Definition of Sports Events Impact Factor

The impact of sports events is generally temporary, and it may cause a spike in traffic flow around the arena. It usually occurs before the start of the game and after the end of the game, which indicates a large number of people entering the vicinity of the arena and a large number of people leaving the vicinity of the arena, respectively. Hence, we can assume that sports events have a weak impact on traffic flow on other days. In this paper, we consider both of the temporal and spatial effects of sports events, since they are also geographically limited.

Based on these assumptions, we use a function with a shape similar to the Gaussian distribution to represent the extent to which sports events affect traffic flow during this special period. In order to make the curve smoother and cover more time periods, we choose the random variable *Z* to obey the Gaussian distribution with mathematical expectation μ and variance Var(Z), which is written as Z∼N(μ,Var(Z)). Furthermore, the probability density function is amplified by Var(Z) to enhance its amplitude and enlarge the influence of impact factor σ. In order to better express the continuous impact on traffic flow before and after the game, a maximum operation is applied to these two distribution functions. Thus, the sports event’s impact factor σ can be calculated as follows:

(7) σ=max[f(z),g(z)]

(8) $$f\left(z\right)=\frac{10}{\sqrt{2\pi }}\exp\left(\frac{-{\left[z-\left(t_{start}-\Delta t\right)\right]}^{2}}{200}\right)$$

(9) $$g\left(z\right)=\frac{10}{\sqrt{2\pi }}\exp\left(\frac{-{\left[z-\left(t_{end}+\Delta t\right)\right]}^{2}}{200}\right)$$

where $t_{start}$ is the start time of the game; $t_{end}$ is the end time of the game; *z* is the sampling time point. As most people will choose to enter the stadium ahead of the start time and stay for a while after the game before leaving, we use Δt to represent this situation.

**Figure 1:**
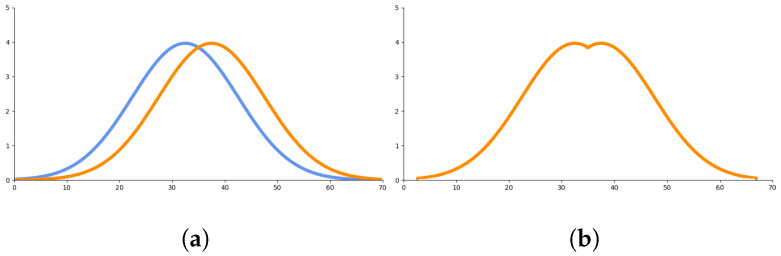
Calculation procedure for σ in one game. (**a**) shows the original f(z) (blue curve) and g(z) (orange curve); (**b**) shows the value σ after taking maximum. σ in this figure only keeps the value between 30 h before the start of the game and 30 h after the end of the game for illustration.

The sports events impact factor σ is illustrated in Figure 1.

Given that the geographical impact of sports events is significant, we introduce a geographical factor β that follows an amplitude-enhanced two-dimensional Gaussian distribution. For node *i*, this factor can be expressed as follows:

(10) $$\beta_{i}=\exp\left(\frac{-[{\left(x_{i}-x_{0}\right)}^{2}+{\left(y_{i}-y_{0}\right)}^{2}]}{2\tau^{2}}\right)$$

where $\left(x_{i},y_{i}\right)$ represents the geographical location of node *i*, and $\left(x_{0},y_{0}\right)$ denotes the arena location where the sports event is held. The *X* and *Y* directions share the same variance $\tau^{2}$.

Under the geographical effect factor β, $\sigma_{i}$ for node *i* can be further derived as follows:

(11) $$\sigma_{i}=\beta_{i}\max\left[f\left(z\right),g\left(z\right)\right]$$

### 3.3. GAT-Informer Architecture

**Figure 2:**
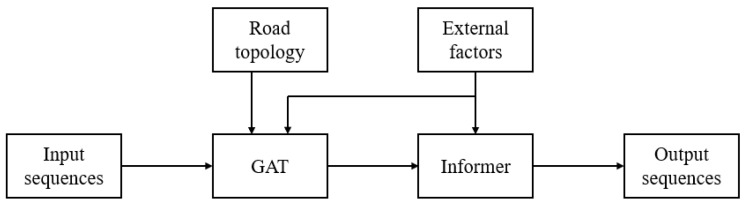
GAT-Informer architecture.

In this subsection, the structure of the GAT-Informer model is introduced in detail. GAT-Informer contains two layers: the Graph Attention layer and the Informer layer. The Graph Attention layer is used to fuse neighbor node information of the input data, so as to capture their spatial features, and then output the time series data with fused neighbor features. The Informer layer is used to process this time series data to achieve long-term prediction. The overview of the GAT-Informer architecture is illustrated in Figure 2. External factors will be introduced into both the GAT layer and the Informer layer for external factor fusion.

#### 3.3.1. Graph Attention Layer

Graph Attention Network (GAT) [40] is a special neural network designed for processing graph-structured data. Unlike traditional neural networks, GAT takes into full consideration the relationships between data during processing, which enables the ability to capture the correlations between data more accurately without manual presetting. When there is a sports event, the adjacency relationships of the traffic network will change, thus rendering the pre-established road adjacency matrix less effective. That is why GAT stands out from other graph neural networks. It is capable of recognizing dynamic node adjacencies and capturing real-time correlations by calculating attention scores for adjacent nodes.

**Figure 3:**
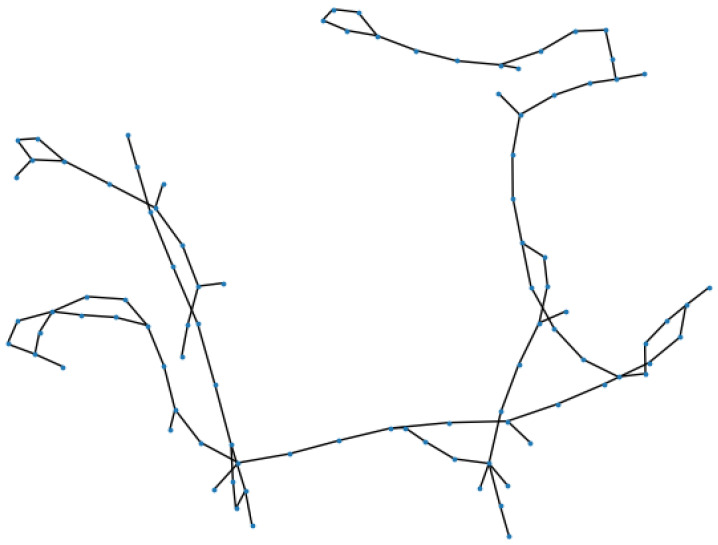
Traffic map.

The input data of the Graph Attention layer contains three parts: a set of node features, the adjacency matrix, and the external impact factor for the consideration of sports events in the spatial information extraction. Node features can be described as an F×n matrix $H=[h_{1},h_{2},\ldots ,h_{n}]$, $h_{i}$∈$R^{F}$, where *n* is the number of detectors in the traffic map, and *F* is the number of input features for each node. Since GAT is a layered strucutre, the input node features of the first GAT layer would be the traffic flow observation *X*. The distance between the detectors can be calculated from the longitude and latitude information in the traffic map, and then the adjacency matrix can be obtained by combining the connection relationship between them. The traffic map with spatial connectivity of the detectors is shown in Figure 3. This traffic map shows the connectivity and relative position of the nodes, but it does not represent the true spatial layout and shape of the road.

**Figure 4:**
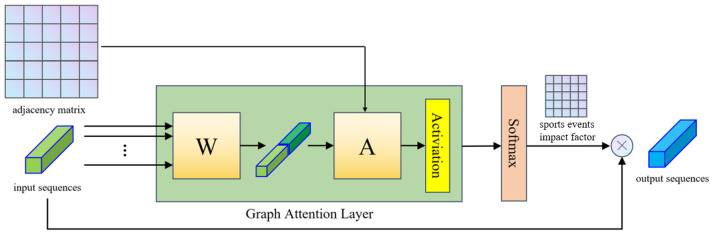
Graph Attention layer architecture.

The entire framework of Graph Attention layer is shown in Figure 4.

As discussed above, the input to this layer contains an F×n matrix (set of node features) and an n×n matrix (adjacency matrix). W and A are both linear transformation matrices with shared parameters and are used for dimension transform. For node *i* and the neighbouring node *j*, attention score $e_{ij}$ can be calculated as follows:

(12) $$e_{ij}=\frac{\exp(LeakyReLU(A(\left[W\cdot h_{i}\right]\left|\right|\left[W\cdot h_{j}\right])))}{\sum_{k\in N_{i}}\exp(LeakyReLU(A(\left[W\cdot h_{i}\right]\left|\right|\left[W\cdot h_{k}\right])))}$$

where || means concatenation. $N_{i}$ refers to neighboring nodes for node *i*.

Although the adjacency matrix is not explicitly present in the core formulas, its role is implicitly crucial. The adjacency matrix defines the relationships between nodes, and only adjcent nodes would be considered when calculating $e_{ij}$. In this paper, we employ a layered strucutre of GAT to perform feature aggregation, and each layer of GAT aggregates information from the direct neighbors of the nodes.

The aggregation of node features is enhanced using a multihead attention mechanism and the sports events impact factor σ. Final output of node *i* can be expressed as follows:

(13) $$h_{i}^{\prime }=\varphi \left(\frac{1}{K}\sum_{k=1}^{K}\sum_{j\in N_{i}}e_{ij}^{k}W^{k}\left(h_{j}+\bar{h_{j}}\sigma_{j}\right)\right)$$

where $e_{ij}^{k}$ are normalized attention scores for the *k*th head of attention. $W^{k}$ is the corresponding linear input transformation, and φ(x) denotes a nonlinear activation function. $\bar{h_{j}}$ refers to the average value of $h_{j}$.

During the feature aggregation, signal from nodes affected by sports events would be enhanced on the basis of their distance to the arena. Final output matrix $\tilde{H}$ remains in the form F×n and contains all aggregated nodes.

#### 3.3.2. Informer Layer

**Figure 5:**
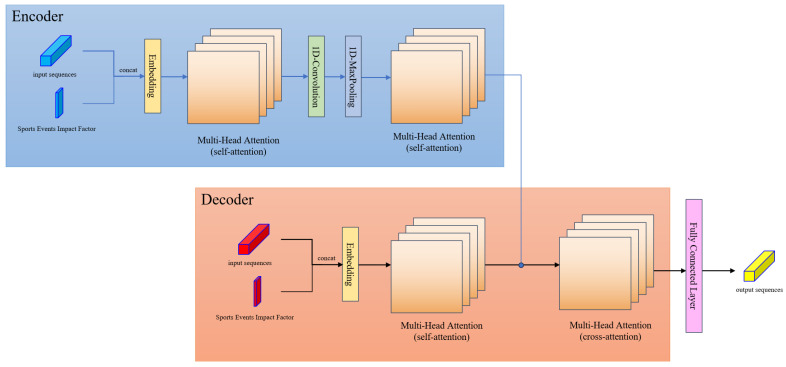
Informer layer architecture. The blue input sequences are the input of encoder, which are the same as the output of Graph Attention layer. The red input sequences are the input of decoder, and they contain two parts: label data and predict data.

Informer is a variant of the Transformer, which achieves higher efficiency than the Transformer in long-sequence time series forecasting [41]. In the scenario of traffic prediction considering sports events, the foreacasting needs to cover a long time span, and the Informer is famous for its ability of long-term prediction and a lower computational cost compared to the Transformer, which enables it to better capture the temporal dependency in a longer prediction horizon. The Informer has an encoder–decoder structure, and the architecture of the Informer layer is shown in Figure 5. The input of the encoder contains two parts: one is the same sequences as the output sequences from the Graph Attention layer $\tilde{H}$; the other one is the sports events impact factor defined in Section 3.2. For node *i*, the time series impact factor $\sigma_{i}$ would be regarded as a temporal indicator and concatenated with the Informer input sequence $h_{i}\in R^{M}$, which can be expressed as follows:

(14) $$h_{i}^{*}=\left[h_{i}|\sigma_{i}\right]$$

By concatenating these two together, each time period will be given a label indicating whether the traffic flow was affected by sports events at that time and the extent to which it was affected. The input of the decoder is similar to the input of the encoder; it also consists of two parts: the main difference is that the input sequences are no longer taken exclusively from the dataset but contain a portion of the data to be predicted, which are initialized by 0. The advantage of this configuration is that the real data in the dataset can guide the predict data, thus improving the accuracy of prediction. The main function of the Informer layer is to assign values to these predict data to obtain the output sequences.

The core idea of the Informer layer is a ProbSparse attention mechanism. The general attention mechanism can be expressed as follows:

(15) $$A\left(Q,K,V\right)=\mathrm{Softmax}\left(\frac{Q\cdot K^{T}}{\sqrt{d}}\right)\cdot V$$

where Q denotes query, K denotes key, V denotes value, d is the dimension of K.

In order to reduce time complexity of this algorithm, ProbSparse attention mechanism optimizes the choice of Q and K. Due to the potential sparsity of the probability distribution of self-attention, only a small number of dot products contribute to the primary attention. Hence, Q is sampled by keeping the active ones and replacing the others with uniform distribution; then, Equation (15) will be updated as follows:

(16) $$A\left(Q,K,V\right)=\mathrm{Softmax}\left(\frac{\hat{Q}\cdot K^{T}}{\sqrt{d}}\right)\cdot V$$

where $\hat{Q}$ has the same size as Q, but we replace the inactive values with mean values.

ProbSparse attention mechanism is only used in self-attention in the encoder. For self-attention in the decoder, since the input sequences contain the data need to be predicted, and due to the prediction causal, future values cannot be used to calculate current values; this will result in the original number of Q and K being already reduced. Hence, there is no need to do further sampling.

The encoder has two layers and contains two self-attention blocks. It forms a basic unit of attention–convolution–maxpooling–attention. If the input sequence is excessively lengthy, it can be extended by adding multiple groups of convolution–maxpooling–attention units after the last attention block. This can effectively reduce the feature dimensions and avoid overfitting. The decoder has one layer and contains only one self-attention block. The output of this block will conduct a crossattention with the output of the encoder.

#### 3.3.3. Loss Function

In order to minimize the error during model training, MSE loss is used as the loss function, which can be written as follows:

(17) $$L\left(pred,true\right)=\frac{1}{n}*\Sigma {\left(pred-true\right)}^{2}$$

where *n* is the number of samples.

## 4. Experiment and Analysis

Our GAT-Informer model was evaluated on two datasets: one is the PeMS04 dataset, which is widely used for traffic flow prediction, and the other one is a newly collected London dataset. We chose five models commonly used for time series prediction for performance comparison. In evaluation using the PeMS04 dataset, the impact of sports events on traffic flow will be disregarded; the target is to compare the performance of these models on solving time series prediction problem. In the London dataset, the sports events impact factor will be included.

### 4.1. Dataset and Preprocessing

#### 4.1.1. PeMS04 Dataset

The PeMS04 dataset records data from 307 detectors on 29 roads in San Francisco Bay Area collected every 5 min between 1 January 2018 and 28 February 2018. The shape of the data is (16,992, 307, 3),where the three-dimensional features are flow, occupy, and speed. Only the flow feature is used in our experiments, thus constructing a (16,992, 307) traffic flow sequence.

#### 4.1.2. London Dataset

**Figure 6:**
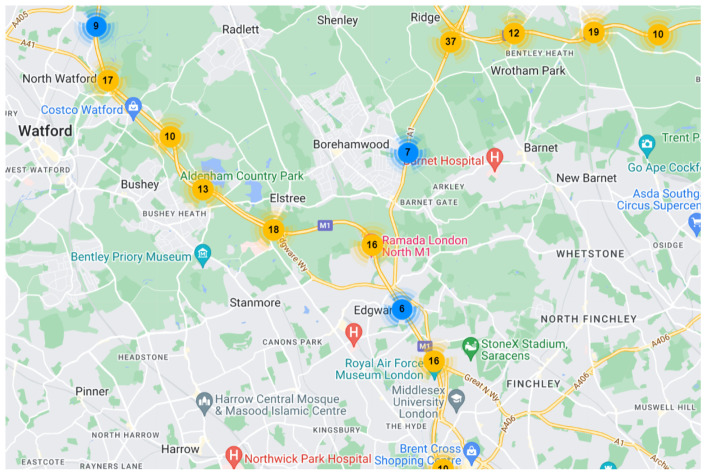
Part of M1 motorway.

**Figure 7:**
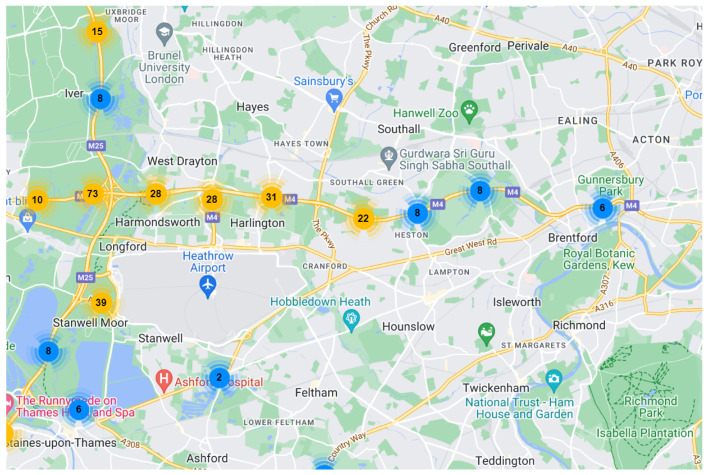
Part of M4 motorway and M25 motorway.

The London dataset records data from 96 detectors on three motorways around London collected every 15 min between 1 August 2022 and 31 October 2022. These three motorways contain M1, M4, and M25. The M1 motorway is a major north–south motorway in England, which connects London and Leeds. The M4 motorway is one of the most important motorways in the United Kingdom, thus running from west London to southwest Wales. The M25 motorway, also known as the London Orbital Motorway, is a ring-shaped high-speed road that encircles London. In order to better study the impact of sports events on traffic flow, the section of M4 motorway and M25 motorway around London Heathrow Airport were selected. The location of detectors used in this dataset is shown in Figure 6 and Figure 7. The dataset contains a (8740, 96) traffic flow sequence.

**Figure 8:**
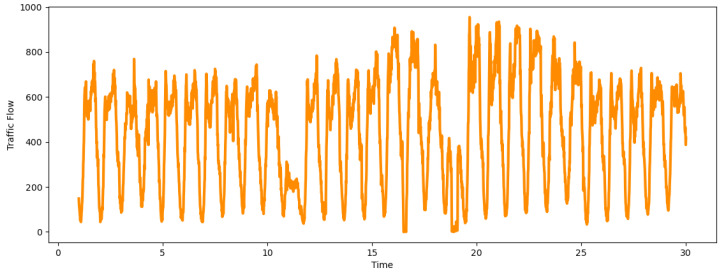
Traffic flow data recorded on M4/2178A from 1 September 2022 to 30 September 2022.

The Laver Cup was held from 23 September 2022 to 25 September 2022 in the O2 arena in London, and this match gained a high level of global attention. Our dataset covers this time period to study the impact that sports events have on traffic flow. Figure 8 shows the traffic flow data recorded by our 2178A detector on the M4 motorway between 1 September 2022 and 30 September 2022. Obviously, there was an uptick in traffic flow around match days compared to other times of this month. Based on the definition in Section 3.2, the sports events impact factor will be calculated for this special period and used as the input of our GAT-Informer model.

#### 4.1.3. Data Preprocessing

Both the PeMS04 dataset and London dataset were min-max normalized in order to accelerate the model convergence, avoid numerical instability, and enhance the model performance. The normalization can be expressed as follows:

(18) $$f\left(X\right)=\frac{X-\min(X)}{\max(X)-\min(X)}$$

### 4.2. Experimental Verification

#### 4.2.1. Evaluation Metrics

Three commonly used metrics were chosen to evaluate the model performance during model training, including the Root Mean Squared Error (RMSE), the Mean Absolute Error (MAE), and the Mean Absolute Percentage Error (MAPE). Each of the metrics is defined as follows:

(19) $$RMSE=\sqrt{\frac{1}{n}\sum_{i=1}^{n}{\left(y_{i}-{\hat{y}}_{i}\right)}^{2}}$$

(20) $$MAE=\frac{1}{n}\sum_{i=1}^{n}\left|y_{i}-{\hat{y}}_{i}\right|$$

(21) $$MAPE=\frac{1}{n}\sum_{i=1}^{n}\left|\frac{y_{i}-{\hat{y}}_{i}}{y_{i}}\right|\times 100$$

where $y_{i}$ and ${\hat{y}}_{i}$ represent the true traffic flow and the predicted one at time *i*, respectively; *n* is the prediction length; Y is the set of $y_{i}$; and $\bar{Y}$ is the average of Y.

#### 4.2.2. Experiment Settings

In the experiment, the collected data were divided into 60% for training, 20% for evaluation, and the remaining 20% for testing. For spatial information extraction, two layers of GAT with three heads of multihead attention were used. For temporal information extraction, three layers of encoder and one layer of decoder with eight heads of multihead attention were employed. The loss function of the MSE was utilized and the loss backwards from decoder outputs to the entire network. The batch size was set to be 32, and the model was optimized using the Adam optimizer with a dropout rate being set to 0.05. The learning rate was adapted from $e^{-4}$, and the number of total training epochs was 50 with early stopping. The experiments were conducted on a computer with an Intel Core i7-12800H 2.40 GHz CPU (Santa Clara, CA, USA) and a RTX 3060 GPU (NVIDIA, Santa Clara, CA, USA). During the experiment, our proposed model will be evaluated across different prediction horizons, together with baseline models, in order to have a comprehensive evaluation in short-term prediction and long-term prediction.

#### 4.2.3. Experimental Results

The traffic flow prediction can be viewed as a time series forecasting problem containing multiple features. For the PeMS04 dataset, we selected five baseline models, and these models have been proven to perform well in dealing with time series problems.

- **GRU**: The GRU is a variant model of RNN. It has been proven to be effective in the short-term time series forecasting problems, and the model also stands out for its ability to alleviate the gradient explosion and vanishing problem. However, the GRU is less effective in the scenario of long-term prediction.
- **T-GCN**: The Integrated GCN and GRU capture both spatial and temporal correlation, and they can be used for both short-term and long-term traffic prediction [42]. However, similar to recurrent-based temporal information, their extraction capacity is weak in long-term prediction.
- **Informer**: The Informer model addresses the computational cost and memory usage problem of the Transformer. The model is widely used in short-term or long-term traffic prediction, but it cannot extract information from adjacent nodes.
- **ASTGCN**: The ASTGCN model is designed for traffic prediction that excels in capturing spatial and temporal dependencies [35]. The novel spatial–temporal attention mechanism enables the model to achieve high performance in traffic prediction. However, this model is constrained by the static map and cannot incorporate external factors.
- **ASTGNN**: The ASTGNN model is an attention-based spatial–temporal model, which stands out for the novel design of a special self-attention mechanism [43]. In both of the short-term and long-term prediction scenarios, the model can achieve satisfying performance.

We deployed the above baseline models to predict traffic flow data for the next 15 min, 30 min, and 45 min horizons based on the 15 min time interval of the PeMS04 dataset. The experimental results are shown in Table 1.

In the 15 min horizon, all the metrics of T-GCN were slightly better than Informer, thus indicating the necessity of graph neural network in solving the traffic flow prediction problem. At the same time, although the T-GCN performed better than the Informer in short-term predictions, its advantage could not be sustained in long-term predictions. This highlights the effectiveness of multihead attention in long-term prediction scenarios. In the 45 min horizon, the RMSE of the GAT-Informer was 13.40% smaller, the MAE was 12.94% smaller, and the MAPE was 14.54% smaller than the ASTGNN, which outperformed most of the baseline models. Moreover, as the prediction horizon lengthened, the performance of models experienced varying degrees of decay. Compared to other baseline models, the proposed GAT-Informer exhibited the least decay, thus maintaining high prediction performance. The MAE of the GRU increased by 10.48% from a 15 min prediction to 45 min prediction, while the MAE of the GAT-Informer only incresed by about 6.56%. However, the difference between the performance of the GAT-Informer and the ASTGCN is not obvious in the PeMS04 dataset experiment.

Based on the above experimental results, the performance of THe Informer, ASTGCN, and the proposed GAT-Informer were evaluated on the London dataset. They were used to predict the future traffic flow data of 15 min, 30 min, 45 min, 60 min, 90 min, and 120 min based on the 15 min time interval. For the impact factor of the sports event, Δt defined in Equations (8) and (9) was set to 0.5 h, and $t_{start}$ and $t_{end}$ followed the schedule of the Laver Cup held in 2022, London. Sports events will affect nodes within a 10-kilometer radius. The experimental results are shown in Table 2.

According to the evaluation metrics in Table 2, GAT-Informer maintained the best performance. In long-term (45 min, 60 min, 90 min, and 120 min) traffic flow prediction, GAT-Informer’s performance was only marginally attenuated; the model still maintained a high fitting degree and had the smallest error between the predict values and true values among all the baseline models. When the input of the model removed the sports events impact factor, the performance of the model decreased. In the 15 min horizon, regardless of the impact of sports events, the RMSE increased by 1.95%, the MAE increased by 6.35%, and the MAPE increased by 6.35%. In the 120 min horizon, regardless of the impact of sports events, the RMSE increased by 1.31%, the MAE increased by 1.02%, and the MAPE increased by 1.03%. Based on the model evaluation, the fusion of sports events is proven to be effective in both short-term prediction or long-term prediction. Although the model performance deteriorated with longer prediction horizons, our proposed model demonstrated the least performance degradation. Meanwhile, the GAT-Informer model considering sports events outperformed the ASTGCN in both short-term and long-term predictions, despite both models achieving similar performance on the PeMS04 dataset. Compared to other baseline models, the proposed GAT-Informer model demonstrates superior capability in extracting information from sports events, thereby enhancing prediction performance and maintaining high prediction accuracy when the prediction horizon lengthens.

#### 4.2.4. Evaluation of Arrival and Depature Redundancy

A redundancy Δt was set in Equations (8) and (9) to represent the early arrival and delayed departure of the audience. Although the start and end times of a game are predetermined, varying redundancies of Δt can influence the impact factor curve, thereby affecting traffic flow predictions. In this case study, we evaluated the effect of different Δt values on the prediction performance of GAT-Informer. The model was evaluated in prediction horizons of 30 min, 60 min, 90 min, and 120 min, with Δt varying from 10 min to 40 min, and the experiment results are shown in Table 3.

Table 3 shows that varying the redundancy Δt did not lead to significant performance changes. However, in general, a redundancy of 20 min slightly improved the prediction performance compared to other settings. This suggests that the impact factor σ influences the model based on the audience arrival and departure patterns. Among different settings, a redundancy of 20 min best represents these patterns in the London dataset.

#### 4.2.5. Results Visualization

**Figure 9:**
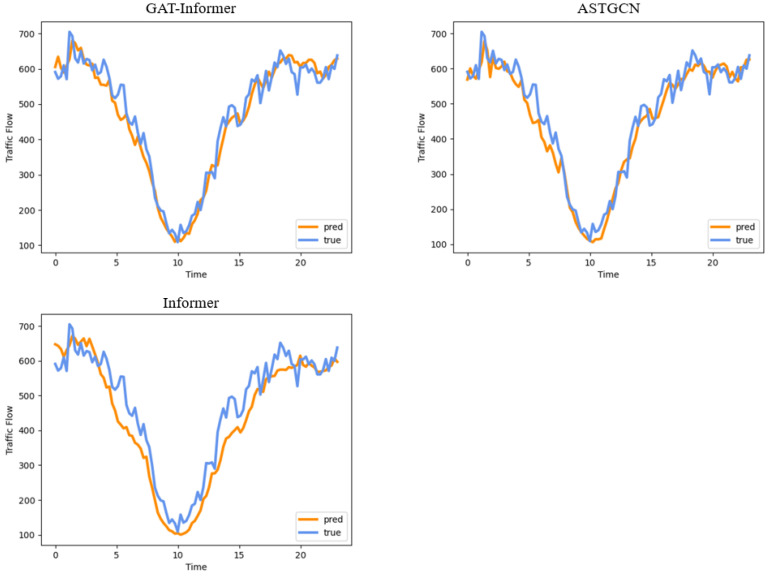
The 15 min horizon: 24 h prediction results on a randomly selected detector.

**Figure 10:**
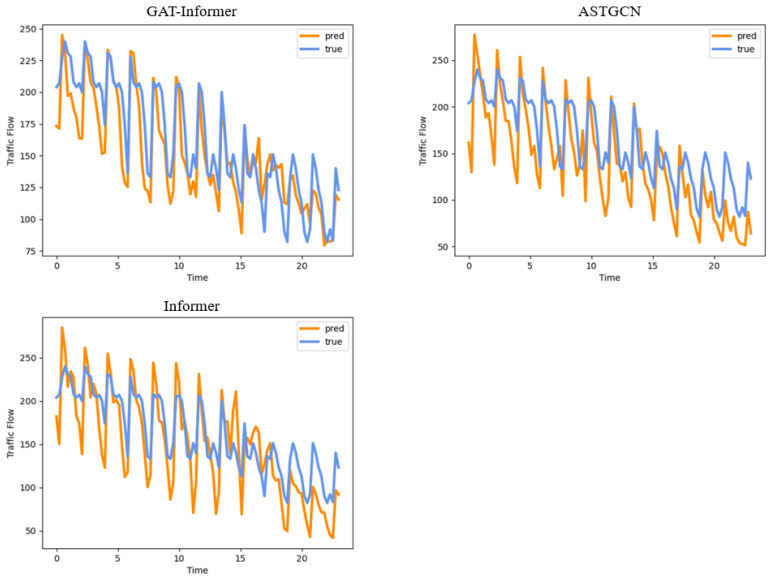
The 120 min horizon: 24 h prediction results on a randomly selected detector.

In order to visualize the prediction performance of each model in Table 2, we randomly selected the prediction results of a continuous 24 h time interval by two different detectors based on a 15 min horizon and a 120 min horizon. The results are shown in Figure 9 and Figure 10, respectively. By comparing the difference between the true values and the predicted values, we can intuitively compare the performance of these three models in terms of short-term prediction and long-term prediction.

From these curves, we can find that our GAT-Informer model had a very good model fit, with a quite small error between the true values and predicted values, thus keeping basically the same curve when dealing with short-term traffic flow prediction. When dealing with long-term traffic flow prediction, although GAT-Informer did not perform as well as the short-term, it was still better than the other baseline models. Its prediction accuracy of peak traffic flow was good, but it had some errors in valley traffic flow. For traffic management, it is often more important to control the peak traffic flow, which may have a greater impact on vehicle travel time.

## 5. Conclusions

In this paper, the GAT-Informer model was proposed for long-term traffic flow prediction considering the impact of sports events. The model contains the Graph Attention layer and Informer layer. Graph Attention layer was used to fuse the information of neighbor nodes so as to fully captured the spatial features. The Informer layer was used to capture the dynamic time correlation between different times based on its ProbSparse attention mechanism. The GAT-Informer and other baseline models were tested on the PeMS04 dataset and London dataset. The experiment results showed that the GAT-Informer had better prediction performance in different horizons and metrics.

The detectors may fail temporarily due to failure, blackout, or other reasons; hence, the traffic flow data recorded by them may have missing data. Future plans include introducing fuzzy representation into the deep learning model to reduce the impact of data uncertainty and thus improve the matching of the model to various datasets.

## Figures and Tables

**Table 1:** Average performance comparison of different baseline models for traffic flow prediction on PeMS04 dataset.

Horizon	Criteria	GRU	T-GCN	Informer	ASTGNN	ASTGCN	GAT + Informer
15 min	MAE	28.04	23.69	26.27	25.69	22.40	**21.78**
	RMSE	45.41	39.42	41.51	40.73	**35.11**	35.24
	MAPE	20.62	17.42	19.32	18.89	16.47	**16.01**
30 min	MAE	30.07	28.90	28.52	27.02	23.05	**22.62**
	RMSE	47.96	44.76	46.40	41.49	35.87	**35.36**
	MAPE	21.82	20.34	20.14	19.23	17.00	**16.98**
45 min	MAE	30.98	30.21	29.44	27.81	**23.91**	24.21
	RMSE	48.94	45.46	43.46	42.51	**36.42**	36.81
	MAPE	22.11	21.25	20.97	19.87	16.95	**16.63**

**Table 2:** Average performance comparison of different baseline models for traffic flow prediction on London dataset.

Horizon	Criteria	Informer	ASTGCN	GAT + Informer	GAT + Informer *
15 min	RMSE	82.61	80.29	**75.86**	77.34
	MAE	48.72	45.42	**42.19**	44.87
	MAPE	12.18	11.36	**10.55**	11.22
30 min	RMSE	86.21	84.65	**81.45**	83.56
	MAE	53.65	49.98	**46.22**	48.08
	MAPE	13.41	12.50	**11.56**	12.02
45 min	RMSE	92.07	87.65	**85.20**	89.77
	MAE	55.80	53.37	**51.52**	53.40
	MAPE	13.95	13.34	**12.88**	13.35
60 min	RMSE	94.83	93.82	**89.82**	91.26
	MAE	60.74	58.27	**56.01**	57.71
	MAPE	15.19	14.57	**14.00**	14.43
90 min	RMSE	112.96	104.16	**100.63**	103.09
	MAE	72.44	65.46	**62.63**	63.17
	MAPE	18.11	16.37	**15.66**	15.79
120 min	RMSE	117.88	107.06	**105.30**	106.68
	MAE	76.11	67.37	**65.85**	66.52
	MAPE	19.03	16.84	**16.46**	16.63

* means the dataset removes the sports events impact factor.

**Table 3:** Average performance comparison of different redundancy Δt values.

Horizon	Criteria	Δt= 10 min	Δt= 20 min	Δt= 30 min	Δt= 40 min
30 min	MAE	49.25	48.46	**48.08**	49.09
	RMSE	84.98	84.40	**83.56**	84.65
	MAPE	12.31	12.12	**12.02**	12.27
60 min	MAE	56.20	56.51	57.71	**56.35**
	RMSE	92.11	91.39	**91.26**	92.17
	MAPE	14.05	14.13	14.43	**14.09**
90 min	MAE	64.04	**63.12**	63.17	64.20
	RMSE	100.88	**100.52**	103.09	101.02
	MAPE	16.01	**15.78**	15.79	16.05
120 min	MAE	68.87	**65.87**	66.52	66.62
	RMSE	109.05	**106.81**	105.30	106.66
	MAPE	18.58	**16.47**	16.63	16.66

## Data Availability

The dataset used in this paper can be found at https://webtris.highwaysengland.co.uk/ (accessed on 25 September 2023).
